# Key Proteins of Replication Stress Response and Cell Cycle Control as Cancer Therapy Targets

**DOI:** 10.3390/ijms25021263

**Published:** 2024-01-19

**Authors:** Alvina I. Khamidullina, Yaroslav E. Abramenko, Alexandra V. Bruter, Victor V. Tatarskiy

**Affiliations:** 1 Laboratory of Molecular Oncobiology, Institute of Gene Biology, Russian Academy of Sciences, 34/5 Vavilov Street, 119334 Moscow, Russia; alvina@genebiology.ru (A.I.K.); yaroslav.abramenko.01@gmail.com (Y.E.A.); 2 Center for Precision Genome Editing and Genetic Technologies for Biomedicine, Institute of Gene Biology, Russian Academy of Sciences, 34/5 Vavilov Street, 119334 Moscow, Russia

**Keywords:** replication stress, DNA damage response, ATR, CHK1, PARP, WEE1, PKMYT1, cyclin dependent kinases

## Abstract

Replication stress (RS) is a characteristic state of cancer cells as they tend to exchange precision of replication for fast proliferation and increased genomic instability. To overcome the consequences of improper replication control, malignant cells frequently inactivate parts of their DNA damage response (DDR) pathways (the ATM-CHK2-p53 pathway), while relying on other pathways which help to maintain replication fork stability (ATR-CHK1). This creates a dependency on the remaining DDR pathways, vulnerability to further destabilization of replication and synthetic lethality of DDR inhibitors with common oncogenic alterations such as mutations of *TP53*, *RB1*, *ATM*, amplifications of *MYC*, *CCNE1* and others. The response to RS is normally limited by coordination of cell cycle, transcription and replication. Inhibition of WEE1 and PKMYT1 kinases, which prevent unscheduled mitosis entry, leads to fragility of under-replicated sites. Recent evidence also shows that inhibition of Cyclin-dependent kinases (CDKs), such as CDK4/6, CDK2, CDK8/19 and CDK12/13 can contribute to RS through disruption of DNA repair and replication control. Here, we review the main causes of RS in cancers as well as main therapeutic targets—ATR, CHK1, PARP and their inhibitors.

## 1. Introduction

Dysregulation of proliferation is the most recognized aspect of cancer. Tumors increase their proliferation rate through increased pro-proliferative signaling and evasion of growth suppressors. As part of these changes tumors lose tumor suppressors and activate oncogenes controlling the entry into the cell cycle, progression to replication and mitosis, and checkpoints, which monitor preparedness for these processes. In short, cancers can risk a higher rate of errors in replication and mitosis for higher rate of proliferation, which also contributes to genetic instability, required for further oncogenic progression. This state of tolerability towards increased rate of errors, together with a higher pressure on replication machinery, creates a state of so-called replication stress (RS). RS is one of the main causes of genome instability and it has serious implications for cell survival, aging and disease development [1]. DNA RS is a cell state that may be caused by different exogenous and endogenous events occurring during DNA synthesis and resulting from defects in the replicative machinery [2].

The three major sources of RS in cancers originate from the most important hallmarks of cancer—the demand for higher proliferation rate, genetic instability and dysregulation of transcription [2,3]. Increased oncogenic activity of pro-proliferative proteins such as Cyclin E, K-Ras, Myc and inactivation of tumor suppressors like p53 and Rb (retinoblastoma protein), leads to decrease of replisome activity, aberrant origin firing [4], and insufficient levels of dNTPs [5] required for replication. Genetic instability caused by defects in DNA damage response (DDR) pathways such as mutations and deregulation of p53, ATM and others leads to collapse of stalled replication forks. Transcription dysregulation caused by activation of Myc and CDK2/Cyclin E, and inactivation of ATR-CHK1 leads to transcription-replication conflicts [6,7].

However, by repressing certain DDR genes, tumors became strongly dependent on remaining DDR branches. Inactivation of the ATM-CHK2-p53 pathway leads to dependency on the remaining ATR-CHK1 DDR, as in the absence of both response pathways DNA repair mechanisms can not be activated, leading to cell death [8]. The concept, when mutations beneficial to the tumor create a vulnerability to inactivation of targets which compensate for such alterations is known as synthetic lethality [9]. For example, RS can be further enhanced by chemotherapy drugs that lead to DNA damage by interfering with replication such as topoisomerase I and II inhibitors, alkylating agents and nucleoside metabolic inhibitors, and tumors that already have high levels of RS are more sensitive to these drugs [10]. Several key studies demonstrated that genetic inactivation of ATR leads to synthetic lethality in *ATM* or *TP53* mutated tumors, paving the way to use of small-molecule ATR inhibitors (ATRi) [11,12]. Similarly other mutations that increase RS through activation of oncogenes or inactivation of tumor suppressors maintaining genome stability leave cancers vulnerable to a whole new class of drugs such as PARP (PARPi) and CHK1 (CHK1i) inhibitors. Another strategy for targeting RS-high tumors is inhibiting activity of proteins which prevent entry into the S-phase and/or mitosis, such as WEE1 and PKMYT1 inhibitors (WEE1i, PKMYT1i), forcing cells into the next phase of the cell cycle, for which they are not prepared. Finally, recent evidence shows that both the cell cycle and transcriptional Cyclin-dependent kinases (CDKs) are implicated in coordinating replication, transcription and DNA repair. Inhibitors of CDK4/6, CDK2, CDK8/19 and CDK12/13 increase RS in a number of models and contribute to their activity shown in clinical trials [7,13,14,15].

This review focuses on such vulnerabilities caused by RS which could be used as targets for cancer therapy as well as markers for such therapies.

## 2. Replication Stress Mechanisms in Cancer

### 2.1. Overview of Replication Control

**Figure 1:**
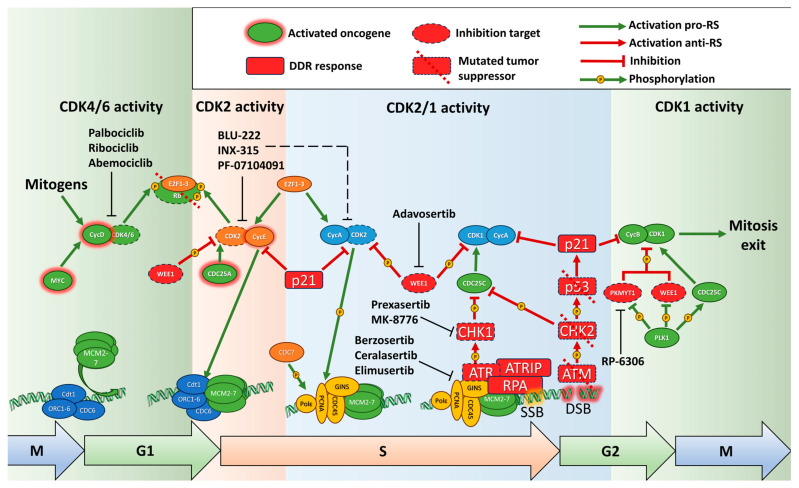
Coordination of cell cycle and replication stress response. Activity of Cyclin-dependent kinases (CDK) in each stage of the cell cycle is critical for stepwise preparation to replication. Pre-replication (MCM) complexes are assembled during late mitosis and G1. Beginning of transcription leads to degradation of Cdt1 preventing further MCM loading onto chromatin. Under conditions of single-strand DNA damage or fork-stalling the ATR-CHK1 response prevents further cell cycle transition. ATM-CHK2-p53 similarly blocks cell cycle after fork-collapse or DNA double-strand breaks. DNA damage response (DDR) and WEE1/PKMYT1 ensure that mitosis does not start before replication is completed. The ATM-CHK2-p53 pathway is frequently mutated in tumors, and inhibition of ATR-CHK1 and WEE1/PKMYT1 compromises remaining response to replication stress, leading to unrepaired DNA damage and cell death.

Replication is a tightly controlled process, which ensures faithful and complete doubling of the DNA. Preparation for replication takes place in the G1 phase, after mitosis or exit from dormancy, and consists of several steps. The origins of replication are recognized by the origin recognition complex, consisting of ORC1-6 proteins, which then recruits CDC6 and Cdt1, which in turn load the replication helicase complex (MCM2-7) onto the chromatin, forming the pre-replication complex (pre-RC). This process is known as “licensing” and prepares points where replication would start [16]. The loading of additional pre-RCs onto the chromatin is prevented by increase of CDKs activity at the initiation of replication [16]. Binding of MCM2-7 induces dissociation of CDC6 and degradation of Cdt1 [17]. The pre-RC complex is then activated by phosphorylation by CDK2 and the Cdc7 kinase, and binding of Cdc45 and GINS in the S phase, forming the pre-initiation complex [18,19]. These events depend on activation of E2F1, after inactivation of Rb by CDK2/Cyclin E [20]. Replication complexes are loaded on chromatin in excessive numbers to ensure complete replication. The excessive, unactivated RC complexes remain dormant in normal conditions, but will start firing in case of the replication fork stalling or collapsing; or the replisome being displaced by the RNA polymerase [21]. Recent evidence suggests that additional RC complexes can be assembled in late S or even G2 phase of the cell cycle to ensure full replication [17]. Coordination of the cell cycle progression, DNA repair and replication completion depends on the replication stress sensor—kinase ATR which slows down replication, prevents origin firing, and activates DNA damage machinery to restart replication forks in case of DNA damage during DNA synthesis. Most importantly ATR activates CHK1 and WEE1 kinases, which in turn inhibit CDKs’ activity, delaying cell cycle progression, especially entry into mitosis [22]. In case the ATR1-CHK1 pathway fails, replication forks would collapse, leading to double-strand breaks (DSBs) and activation of the ATM-CHK2-p53 pathway (Figure 1).

All of the above processes are often altered during oncogenic progression. The impairment of normal replication process, resulting in increased stalling and collapse of replication forks is defined as RS. In the following sections we would discuss how stimulation of oncogene activity and suppression of oncogenes affects the normal process of replication, leading to RS in cancer.

### 2.2. Oncogenic Transformation Leads to Replication Stress

A significant portion of oncogenes deregulated in cancers consists of components of signaling pathways required for entry into the cell cycle from quiescence. the link between dysregulation of proliferation and replication-dependent DNA damage and genetic instability was recognized in the late 1990s–early 2000s [23,24], with experiments that transduced active oncogenes into non-transformed cells. a key early study by Di Micco et al. [25] demonstrated that introduction of activated H-Ras into normal human fibroblasts led to burst of proliferation, followed by increased DDR and subsequent oncogene-induced senescence. DNA damage was associated both with under-replication in the S phase and re-replication due to aberrant origin firing. Knockout of DDR components such as CHK2 prevented cells from exiting the cell cycle and allowed maintenance of proliferation with accumulation of DNA damage. In parallel, induction of pro-survival signaling like ERK in Ras-mutated cancers prevents cell death and p53 activation by DDR [26]. Other mutations which upregulate Ras activity or act downstream of it such as B-Raf [27,28], EGFR [29,30] and others lead to similar effects [4].

Downstream of the signaling pathways is the cell cycle machinery (Figure 1). The proliferation signals converge on increase of *CCND1* transcription. Cyclin D1 (product of *CCND1*) is instrumental in leading the cells into replication through phosphorylation of Rb and transcription and stabilization of E Cyclins. Despite that, overexpression of Cyclin D1 does not induce DDR markers and increase of RS [31].

E Cyclins (E1 (coded by *CCNE1*) and E2 (*CCNE2*), together further referred as Cyclin E) are critical to entry into the S phase, and its deregulation is one of the main causes of RS. E Cyclins are aberrantly expressed in 21% of cancer (data from TCGA Pancancer Studies, accessed through Cbioportal.org [32]) with highest levels in breast, ovarian and endometrial cancers. Cyclin E/CDK2 complex polyphosphorylates Rb, completely inactivating it and releasing the E2F1 transcription factor. Besides that, Cyclin E is involved in licensing the origins of replication and centrosome duplication [33,34]. Cyclin E phosphorylates a number of substrates like CDC6 in the origin recognition complex and other components of the pre-replicative complex—Recql and Treslin, and increases expression of *CDC6*, *CDT1*, required for loading of the pre-RC complex, and *MCM* genes of the pre-RC complex itself (reviewed in [35]). Despite its obvious pro-oncogenic role, Cyclin E, while increasing the entry into the S phase, lowers the speed of replication, because of RS. Excessive amounts of Cyclin E diminishes MCM2-7 loading on chromatin, and induces aberrant firing of origins, causing transcription-replication conflicts [36]. Cyclin E overexpression stimulates pre-initiation complex formation, but inhibits the pre-replication complex and interferes with proper origin firing [35,37]. High levels of Cyclin E1 further lead to chromosome segregation defects and mitotic catastrophe. This effect can be counteracted by further mutations in DDR response [37]. A particularly pro-oncogenic form of Cyclin E—processed low-molecular-weight Cyclin E (LMW-E) increases both entry into the S phase and its speed and has a particularly negative prognosis [38]. LMW-E increases RS tolerance through both increasing pre-replication complex assembly and DNA repair through Rad51 [39]. Notably, LMW-E-expressing tumors are particularly resistant to targeted therapy, as its complex with CDK2 is resistant to CDK inhibitor proteins such as p21 and p27 [40], while CDK2 activity decreases p27 levels through phosphorylation [41]. *CCNE1* amplification is one of the main markers both for synthetic lethality in cancer models [42,43,44] and for use of drugs affecting RS in the clinic [45]. High levels of Cyclin E1 together with high levels of genetic instability can serve as markers for expanding the use of RS targeted drugs beyond breast and ovarian cancers [46]. Besides direct mutations in *CCNE1*, it can be stabilized through inactivating mutations of *FBXW7*, its product is a component of the Skp1-Cullin1-F-box (SCF) E3 ligase, which degrades Cyclin E. Mutations of *FBXW7* additionally increase RS through stabilization of Myc [47].

### 2.3. Transcription-Replication Conflicts Are Increased in Cancers

**Figure 2:**
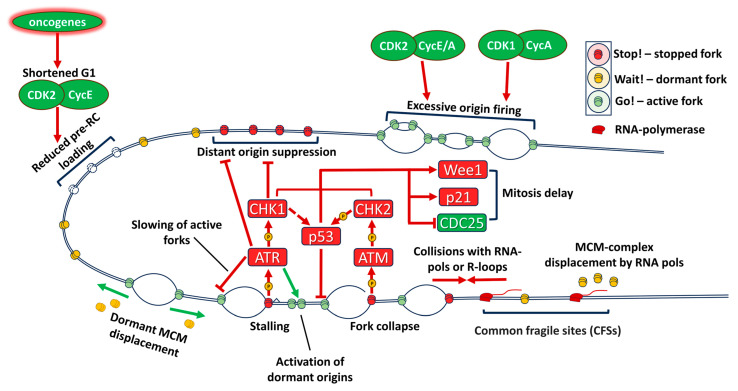
Sources of replication stress in cancer. Increased activity of oncogenes and CDK2 leads to reduced MCM complex loading to chromatin during a shortened G1, preventing complete replication. Increased activity of CDKs during replication activates excessive and aberrant origin firing, depleting dNTPs and RPA proteins and causing re-replication. Deregulated transcription intensifies collisions between RNA polymerases and DNA replication machinery and displaces MCM complexes from transcriptionally active genes. DNA damage response (DDR) is activated by fork stalling and fork collapse and slows active forks, activates nearby dormant origins and blocks distant origins. This ensures orderly completion of replication, while transition to mitosis is prevented by inhibition of CDK A/CyclinB1.

A particular mechanism of elevated RS in cancer is increase of transcription-replication conflicts. The transcription factor c-Myc also acts as a universal amplifier of transcription, increasing transcription rate for thousands of genes [48]. Activated Ras [29] and other oncogenes also increase transcriptional activity. Collisions of the replication machinery with transcription complexes [49] or R-loops formed by the RNA-DNA hybrids [50] lead to fork stalling and collapse, which activate DDR and increasing RS (Figure 2). Under normal conditions transcription and replication are spatially separated, especially in transcriptionally active regions such as nucleoli [51]. To ensure complete replication of transcribed regions, cells must activate the S-phase checkpoint (ATR-CHK1-dependent inhibition of CDK1) to prevent onset of mitosis before end of replication. Deregulation of these mechanisms increases genomic instability [17]. Transcription machinery also can displace the MCM complexes from DNA, so transcribed genes may often conflict with licensed origins, leading to under-replicated DNA [52]. These under replicated sites of active transcription are therefore commonly broken in mitosis and are known as common fragile sites (CFSs). Recent data suggests that new MCM complexes can be assembled in the S phase through stabilization of Cdt1 [17], but this assembly relies on activation of DDR.

### 2.4. Rb/E2F Pathway, Transition to S Phase and Replication Stress

Phosphorylation of Rb and subsequent cell cycle promotion to S phase by release of E2F transcription factors primarily depends on Cyclin D-CDK4/6 activity and after that on p53 and Cyclin E/A-CDK2 [20,53,54]. In vivo experiments have shown that if tumor cells originally have *RB1* (gene of Rb protein) depletion, this Rb status will be obligatory for the further growth and survival. Thus, recovery of intact Rb significantly decreased tumor proliferation after continuous growth and reduced levels of *CCNE2*, *PCNA* and *MCM3* genes’ expression, which is vital for cell cycle progression [55]. Mutations of *RB1* are a major contributor to RS and genetic instability in general. *RB1* is frequently mutated in a number of cancers (7% in total, according to TCGA database), including sarcoma, bladder, endometrial, ovarian, breast, prostate and non-small cell lung (NSCLC) cancers, and the Rb protein is inactivated by high CDK4/6 or CDK2 activity [32,56,57]. Rb mainly contributes to genetic instability through release of E2F1, which leads to accumulation of γH2AX foci and increased phosphorylation of RPA (replication protein A) (Figure 1). Rb-E2F1 complex is required for condensin II (a key player of mitotic chromosome assembly) recruitment, preventing under-replication [58]. Additionally Rb is recruited to stalled replication forks, displacing PCNA and allowing their repair [59]. E2F1 recruits DDR proteins through Rb and its interactions with epigenetic modifiers such as BRG1, p300/CBP and others, maintaining genome stability and preventing RS [60]. Rb has a number of functions apparently independent of the E2F family through an extensive network of protein interactions [61]. One of the studies was devoted to the aberrant activation Rb/E2F by oncogenic proteins E6/E7 encoded by human papillomavirus HPV-16 or Cyclin E that lead to depletion of the nucleotide pool. It caused RS and DNA damage accumulation (DSBs, γH2AX foci), however Myc activated nucleotide biosynthesis that, in turn, restocked nucleotides level, rescuing the phenotype [62]. *RB1* mutations seem to be indicative of sensitivity to inducers of RS, including PARP trapping inhibitors [63]. Combined mutations of *RB1* and *TP53* lead to resistance to any chemotherapeutics, but increased sensitivity to inductors of RS, including ATRi and PARPi [64]. 

### 2.5. Replication in G2 Phase and Transition to Mitosis

WEE1 kinase is one of the most well-known key regulators of mitotic entry. Phosphorylation and subsequent inactivation of CDK1 is vital for G2/M checkpoint [65] and spindle assembly checkpoint [66]. Its antagonist, protein phosphatase CDC25, performs activation of CDK1 [67], while CDC25 activity depends on inhibiting phosphorylation by CHK1 (Figure 1 and Figure 2). Besides, WEE1 has an additive role as a CDK2 inhibitor during replication [68] that is crucial for correct origin firing and replication fork progress. WEE1 deficiency in cancer cells leads to hyperactivation of CDK1 [69] and CDK2 [68], thereby leading to multiple initiation of replication origins that causes depletion of replication factors, subsequent fork slowing, accumulation of single-stranded DNA and genomic instability followed by accumulation of RPA. Furthermore, WEE1 acts as a protector of the replication fork from DNA2 nuclease [70]. 

### 2.6. DNA Damage Response and Replication Stress

Presence of continuous DNA damage in cells with activated oncogenes, led to investigations of precise mechanisms by which dysregulation of cell cycle control and S phase entry leads to genomic instability. DNA damage in general activates either ATM (ataxia telangiectasia mutated) kinase that responds to DSBs or ATR (ataxia telangiectasia and Rad3 related protein) kinase which can be activated by SSBs, as well as chemical adducts and replication fork stalling. They in turn activate CHK1 (checkpoint kinase 1) and CHK2 (checkpoint kinase 2), which block progression through the cell cycle and phosphorylate components of the DNA repair machinery, allowing the cell to reverse the damage, while not propagating it to daughter cells [71] (Figure 1 and Figure 2).

The MRE11–RAD50–NBS1 (MRN) complex serves as a DNA damage sensor that identifies DNA DSB sites and binds them, initiating DNA repair. After autophosphorylation, the MRN complex induces ATM activation that is involved in regulation of the cell cycle via the checkpoint kinase CHK2. ATR is a master regulator of response to DNA single-stranded breaks (SSB), potentiates subsequent RS response and cell cycle block via CHK1. Activation of the ATR/ATM signaling pathways is a key process involved in DDR in the intra-S phase checkpoint leading to cell cycle arrest, DNA repair or apoptosis induction [72]. 

ATM plays a central role in DSB repair and is involved in the response to RS only indirectly. The key role in response to RS is played by the ATR kinase. Firstly, a single strand DNA sensor RPA is activated and binds to the stalled replication forks. Then, together with ATR-interacting protein (ATRIP), RPA recruits ATR kinase that in turn activates DNA topoisomerase II-binding protein 1 (TOPBP1) and phosphorylates CHK1 inducing the ATR-CHK1 pathway. It leads to S/G2 cell cycle arrest through CDK2 reduction and CDK1 phosphorylation by WEE1 and subsequently to DNA repair activation. ATR is a general responder to RS and its inhibitors will be reviewed below. Pharmacological inhibitors of ATR are widely studied as agents of synthetic lethality in the context of *ATM* mutations or deletion, DNA damage inducing drugs or radiotherapy where replicative stress is high. CHK1 is a serine/threonine checkpoint kinase downstream of ATR induced in response to DNA damage and RS and therefore regulates cell cycle mitotic progression. CHK1 is the effector of the intra-S and G2/M phase checkpoints [73]. CHK1 forms a complex together with checkpoint regulatory protein Claspin and is phosphorylated at Ser-345 and Ser-317 by ATR [73,74] or independently of ATR through autophosphorylation. [75,76]. CHK1 facilitates the degradation or sequestration of CDC25A phosphatases that remove the inhibitory phosphorylation of CDKs by WEE1 and allow cell cycle progression. Thus, CHK1 delays cell cycle progression until resolution of RS. Also, CHK1 plays a role in mitotic progression and mitotic exit phosphorylating CDC25 phosphatases, preventing CDK1 activation, and stopping the cell cycle in the G2 phase [73].

There are a number of studies reporting the importance of CHK1 in cancer treatment. It was demonstrated that CHK1 inhibitors exacerbate RS induced by insulin-like growth factor inhibition, inducing cancer cell death through replication catastrophe [77].

PARP family is known as a main regulator of SSB repair [78]. The mechanism consists of binding to DNA and synthesis of poly(ADP-ribose) chains to engage DNA-repairing proteins such as XRCC1 and Pol β. Thus, the PARP family is involved in base excision repair (BER), which is a major pathway in SSB repair. Also there was some evidence that PARP can promote an alternative pathway of non-homologous end joining (NHEJ) called microhomology-mediated end joining (MMEJ) [79,80]. Besides, PARP is also responsible for the avoidance of DSB by preventing transforming of SSB to DSB during DNA replication that can be a significant trigger of genomic instability and RS [81]. Hence, inhibition of PARP in cells with a decreased homologous recombination (HR) level, commonly indicating BRCA1/2 defects, may lead to the inability of SSB and DSB repair and further to programmed cell death [82]. It was demonstrated that the ATR-CHK1 pathway is vital for PARP-mediated response to DNA damage, according to their roles as restrainers of cell cycle progression by inhibition of CDK1 and CDK2 [83].

### 2.7. P53 and RS

P53 as one of the most crucial regulators of different biochemical pathways is predictably engaged in RS occurrence and response. P53 affects response to RS through its activities related to both DNA repair and influence on cell cycle (Figure 1 and Figure 2). The first function consists of p21 induction with subsequent G1/S and G2/M arrests through binding to CDK1 and CDK2, therefore p53 is responsible for Rb phosphorylation and further cell cycle promotion to S phase [84]. Besides, p53 is also known as G2 arrest activator due to its function as an inducer of GADD45 and 14-3-3σ, proteins that impair Cyclin B/CDC2 complex [85,86]. In cells overexpressing *CCNE1* and experiencing high RS, p53 was important for preventing catastrophic mitosis through increased levels of p21, WEE1 activity and activation of APC/C^Cdh1^. This leads to endoreduplication (mitosis bypass, leading to polyploidy) in p53-proficient cells and senescence, which is reversed in *CCNE1*-expressing cells [87]. 

P53 has multiple roles in DNA repair and response to RS. P53 participates in replication of DNA under normal conditions and in RS. Participation in DNA repair processes includes recruitment of 53BP1 protein, which is involved in a NHEJ repair and suppression of *RAD51* gene, which is pivotal for HR, thereby regulating main mechanisms of DSBs repair [88,89]. On the other hand, p53 is involved in SSB repair by modulating *APE1* expression, according to its participation in DNA BER [90]. P53 helps to resolve conflicts between transcription and replication, and its loss is synthetically lethal with inhibition of topoisomerase II [91]. P53 binds to both active and stalled replication forks, helping to restart stalled forks and organize DNA repair enzymes at these sites, activating MRE1 and suppressing error-prone Rad52 and POLθ-mediated repair [92,93]. P53 depletion slows replication fork progression [94], which is dependent on its transcriptional activity. This result explains increased tolerance of p53-proficient cells towards chemotherapeutics that increase RS, including gemcitabine and WEE1i [95,96]. In addition to mutations, p53 inactivation or silencing, through MDM2 hyperexpression or oncogenic activation of Ras, also increases sensitivity to RS-inducing drugs, such as ATR-CHK1 inhibitors [97]. These results point to p53 inactivation as a vulnerability for RS-inducing drugs and as a marker for their clinical use.

## 3. Small Molecule Inducers of Replication Stress

### 3.1. ATR-CHK1 Inhibitors

**Figure 3:**
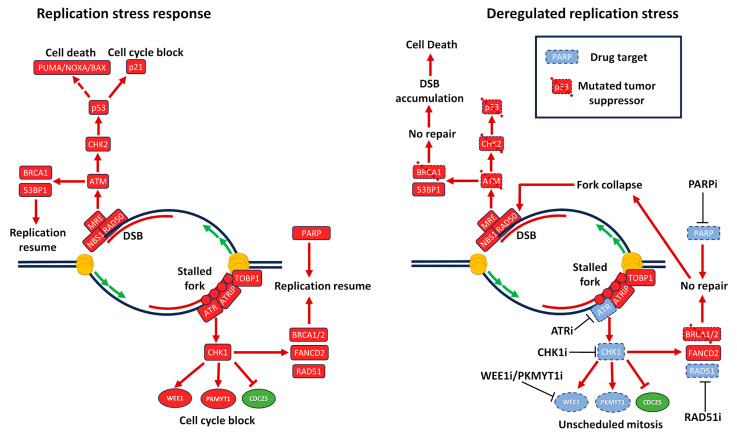
Small-molecule inducers of replication stress. DNA damage during replication induces ATR and ATM responses and DNA repair (homologous recombination through BRCA1/RAD51, fork protection and stabilization through RAD51 or FANC2, non-homologous end joining through though 53BP1, PARP-dependent DNA repair and others). Mutations in ATM-CHK2-p53 reduce response to DSBs, and inhibition of ATR-CHK1, RAD51 and PARP compromises replication fork rescue, causing irreparable replication fork collapse. Inhibition of WEE1 and PKMYT1 prevents replication completion, causing DNA damage in mitosis and cell death through mitotic catastrophe.

The most important function of the ATR–CHK1 signaling pathway is to stabilize replication forks, limit the number of active origins and repair of DSB and collapsed replication forks during S phase. By repressing the origins’ firing the ATR-CHK1 pathway realizes control of the cell cycle and maintains the genomic integrity in response to DNA damage and RS (Figure 3). On the other hand, RS that is common among different types of cancers serves as a potent activator of ATR-CHK1 signaling [98]. Thus, the ATR-CHK1 pathway is considered as an attractive target for anticancer therapy.

ATRi including berzosertib (also known as M6620, VX-970) and gartisertib (M4344, VX-803) produced by Merck KGaA (Darmstadt, Germany), ceralasertib (AZD6738) by AstraZeneca (Cambridge, UK), elimusertib (BAY1895344) by Bayer (Leverkusen, Germany), camonsertib (RP-3500) by Repare Therapeutics Inc. (St-Laurent, QC, Canada), and ART0380 by Artios Pharma (Cambridge, UK), have demonstrated high anti-cancer activity, especially in cancers with high RS level and increased ATR-CHK1 dependency, oncogenic Ras activation, *CCNE1* or *MYC* amplification [99]. Also, small molecule ATRi have been employed to improve the efficacy of DNA damage-based chemotherapy for rapid elimination of proliferating tumor cells. It was demonstrated that ATRi could improve response of cancer cells to conventional chemotherapeutic agents as studied in a rapidly growing number of clinical trials (Table 1).

Berzosertib is a potent and ATP-competitive selective small-molecule ATRi [100]. Berzosertib is currently in 1/2 phase clinical trials in combination with other anticancer treatments, specifically chemotherapeutic drugs. It showed single-agent activity, as well as synergistic activity in combination with cisplatin, especially in advanced cancers with ATM aberrations confirming synthetically lethal interaction between ATM deficiency and ATR inhibition during the phase 1 clinical studies [101,102,103]. Berzosertib is effective in the treatment of brain metastases from NSCLC enhancing the effect of radiation [104], in combination with topoisomerase I inhibitor topotecan in small cell lung cancer [105], and with cisplatin in neuroendocrine tumors [106]. Prominent results were demonstrated on chemotherapy-resistant small cell neuroendocrine cancer and high-grade serous ovarian cancer (HGSOC), which exhibit high levels of RS. Combinations of berzosertib with topoisomerase I inhibitors and gemcitabine were synergistically cytotoxic and showed durable tumor regressions and increase in progression-free survival [105,107,108]. Berzosertib demonstrated synthetic lethality in vitro and in vivo in tumor cells with *ARID1* deletion or mutations, one of genes commonly altered in cancers [100,109,110]. 

AZ20 is a potent and selective ATRi that belongs to sulfonylmorpholinopyrimidines. AZ20 inhibits ATR and ATR-mediated phosphorylation of Chk1 in HT29 colorectal adenocarcinoma tumor cells. AZ20 demonstrated high antiproliferative activity against different neoplasms in vitro and in vivo [111,112,113]. The structure of AZ20 was optimized to create AZD6738 (ceralasertib)—another ATRi with improved preclinical physicochemical and pharmacokinetic characteristics [114,115]. Ceralasertib was active as a single agent in NSCLC cell lines [116] and potentiated the cytotoxicity of cisplatin and gemcitabine in NSCLC cells, and ATM-deficient lung cancer xenografts [117]. Similar synergistic effects were demonstrated for pancreatic ductal adenocarcinoma (PDAC) [118]. Ceralasertib inhibited gemcitabine-induced CHK1 activation and prevented cell-cycle arrest, leading to the strong induction of RS markers. For instance, ATR inhibition by ceralasertib promotes sensitization to cisplatin in head and neck squamous cell carcinoma (HNSCC) regardless of presence of HPV, one of the important causes of oropharyngeal infection [119]. There is a complex interaction between HPV and DDR proteins. HPV creates aberrant DNA structures during its rapid replication thereby activating cellular RS and recruiting DDR proteins. Subsequently, activation of the ATR signaling pathway leads to the DNA repairing, facilitating viral replication and ensuring a successful viral life cycle [120]. Thus, in in vivo studies, combined treatment with cisplatin and ceralasertib exhibited greater anti-tumor effects, relative to either mono agent, against both HPV+ and HPV− xenograft models, including patient-derived xenograft (PDX) models [119]. Anti-tumor activity of ceralasertib as mono agent and with cisplatin was demonstrated on HER2-positive breast cancer in vitro [121].

Sensitivity to ceralasertib is elevated in models with increased RS such as tumors with defects in the ATM pathway or *CCNE1* amplification [122]. Complete loss of ATM function in PDAC models is also critical for efficacy of ATRi/gemcitabine combinational treatment [123]. Ceralasertib also showed increased activity in other models with high genomic instability, such as a *BRCA2*-mutant triple-negative breast cancer (TNBC) PDX model. Moreover, ceralasertib had combinatorial efficacy with chemotherapy medications carboplatin and irinotecan and the PARPi olaparib [122]. Ceralasertib is currently being evaluated in a 1/2 phase clinical trials and one study is in a 3 phase and is aimed to check the efficacy and safety the inhibitor in combination with durvalumab in patients with locally advanced and metastatic NSCLC after progression on prior anti-programmed death ligand 1 (anti-PD-L1) therapy and platinum-based chemotherapy (NCT05450692, Table 1). It has been demonstrated that the DNA damage checkpoint plays a critical role in regulating PD-L1 expression [124]. It was demonstrated that PD-L1 expression in cancer cells is upregulated in response to DSBs and requires ATR/CHK1 kinases [125]. Anti-PD-L1 can be combined with ATR targeted drugs to improve therapeutic response to immune checkpoint blockade therapy. 

Elimusertib (BAY1895344) is a ATR kinase inhibitor in 1/2 phase clinical trials that showed synergistic antitumor activity in combination with DNA damage-inducing, repair-compromising chemotherapy or radiotherapy in preclinical cancer models. Moreover, it improved antitumor efficacy of nonsteroidal androgen receptor antagonist darolutamide in hormone-dependent prostate cancer [126]. High-risk neuroblastomas are in a group of tumors with oncogene-induced RS because of *MYCN* amplification and frequent *ALK* mutations. It was shown that elimusertib, together with ALK inhibitor, potently inhibited cell growth, and led to complete tumor regression in mice models [127]. Elimusertib demonstrated high efficacy against aggressive uterine leiomyosarcoma harboring ATRX mutations in in vivo models [128] and ovarian and uterine carcinosarcoma cell lines and xenografts [129]. Together with PARPi, elimusertib potentiated antitumor activity of HER2-targeted antibody-drug conjugates in HER2-positive cancer in vitro and in xenograft models [130]. Treatment by elimusertib together with anti-PD-L1 resulted in high antitumor activity in a syngeneic mice model with androgen-indifferent, aggressive prostate cancer [131]. The inhibitor is currently in 1/2 phase clinical trials (Table 1).

Gartisertib (M4344, VX-803) is a relatively novel ATP-competitive ATRi that is currently in clinical development. Gartisertib suppressed cancer cell proliferation at concentrations similar to elimusertib and was more potent than berzosertib and ceralasertib [132]. It, as other ATRi, is highly synergistic with a broad range of DNA-targeting anticancer agents including topoisomerase inhibitors, gemcitabine, cisplatin, and PARPi [132,133]. The anticancer activity of gartisertib was demonstrated in multiple cancer cell lines, patient-derived tumor organoids, and mouse xenograft models. In combination with ATM inhibitor M4076, gartisertib demonstrated high anti-tumor efficacy in PDX models of TNBC [134]. The inhibitor is in 1/2 phase clinical trials (Table 1).

Camonsertib (RP-3500) is a 1/2 phase clinical-stage [135] inhibitor that demonstrated high effectiveness in preclinical models as a monotherapy and in combination with PARPi olaparib or niraparib [136]. Another novel ATRi—ART0380—is in a phase 1/2 clinical trial as a monotherapy or in combination with gemcitabine in patients with advanced or metastatic solid tumors (Table 1).

ATR inhibition is effective in combination with other drugs that induce RS. For example, ATR inhibition by ceralasertib as a single agent and in combination with either CHK1 or WEE1 inhibitors was effective in several preclinical models of Mantle cell lymphoma and diffuse large B-cell lymphoma (DLBCL) regardless of their *TP53*, *MYC*, and *ATM* mutational status in vitro and in vivo studies [137]. Also high antitumor activity of ATRi (ceralasertib) and WEE1i (adavosertib) was shown in non-germinal center DLBCL cell lines, characterized by high *MYC* expression and *CDKN2A/B* deletion [138]. ATR inhibition led to accumulation of 53BP1 nuclear bodies in daughter G1 cells and G1 arrest. WEE1 inhibition caused more pronounced DNA damage, inducing arrest in the S phase, and rapid induction of apoptosis. In vivo xenograft DLBCL models showed potential for effective ATRi combinations. Moreover, ATRi and CHK1i are shown to resensitize PARPi-resistant, *BRCA1*-deficient cancer cells to PARPi [139,140,141,142,143] that also makes ATR-CHK1 pathway an attractive target in drug resistance context. 

Particular challenge for anticancer therapy is to kill cells in a quiescent or slowly growing state. So it was demonstrated that treatment with the ATRi enhanced cell apoptotic signaling induced by cisplatin in quiescent cancer cells in vitro [144].

**Table 1:** Current clinical trials of ATRi.

Compound	Study Phases	Key Indications	References
Berzosertib (M6620, VX-970)	Phase 2 (7 trials)	Different types of cancer, including DDR deficient and *TP53* mutant tumors	NCT02595892 [107] NCT04266912 NCT03517969 NCT02567409 [145] NCT03896503 NCT04216316 NCT03641313 NCT03718091 (completed) NCT02487095 [146,147]
	Phase 1 (9 trials)	Different types of cancer, including DDR solid tumors	NCT02723864 NCT02589522 NCT02595931 NCT05246111 NCT04266912 NCT02567422 NCT02627443 NCT04216316 NCT04052555 NCT02157792 [101,102,103,108]
Ceralasertib (AZD 6738)		NSCLC	NCT05450692
	Phase 2 (28 trials)	Different types of solid tumors, including NSCLC, breast and ovarian cancers	NCT02264678 NCT04417062 NCT05061134 NCT05941897 NCT04564027 NCT05582538 NCT03801369 NCT04699838 NCT04090567 NCT03579316 NCT03878095 (suspended) NCT04239014 (withdrawn) NCT03334617 NCT03330847 NCT02937818 NCT03833440 NCT02813135 NCT04298021 NCT04298008 NCT03462342 NCT03428607 (completed) NCT03780608 NCT04065269 NCT04361825 NCT02576444 (terminated) NCT03740893 NCT03182634 NCT02664935
	Phase 1 (13 trials)	Different types of solid tumors and leukemias	NCT05469919 NCT02264678 NCT05514132 NCT03328273 NCT03022409 (completed) NCT04704661 NCT03669601 NCT02630199 NCT03770429 NCT02223923 NCT01955668 (completed) NCT03527147 (completed)
Elimusertib (BAY1895344)	Phase 2 (1 trial)	Relapsed or refractory solid tumors	NCT05071209
	Phase 1 (10 trials)	Different types of carcinomas and lymphomas	NCT05010096 (withdrawn) NCT03188965 (completed) NCT04095273 (completed) NCT05071209 NCT04616534 NCT04267939 NCT04491942 NCT04535401 NCT04576091 NCT04514497
Gartisertib (M4344, VX-803)	Phase 2 (1 trial)	Advanced breast cancer with DDR mutations	NCT04655183 (withdrawn)
	Phase 1 (1 trial)	Solid tumors	NCT02278250 (completed)
Camonsertib (RP-3500)	Phase 1/2 (2 trials)	Advanced solid tumors	NCT04972110 NCT04497116
ART0380	Phase 2 (1 trial)	Advanced tumors	NCT05798611
	Phase 1/2 (1 trial)	Advanced tumors	NCT04657068

CHK1 inhibitors (CHK1i) have actively been investigated in different tumor models and in combinations with a variety of drugs. The most promising compounds are prexasertib by Eli Lilly & Co. (Indianapolis, IN, USA), SCH 900776 by Merck and Co. (Rahway, NJ, USA), and SRA737 by Sierra Oncology Inc. (San Mateo, CA, USA) all of which are in early clinical trials (Table 2).

Prexasertib (LY2606368, ACR-368) is a highly selective dual CHK1/CHK2 inhibitor that prevents CHK1 autophosphorylation, stabilizing CDC25A and increasing RS, leading to replication catastrophe and apoptosis [148] (Figure 3). It is effective in monotherapy and in combination with other replication-stress inducing agents such as PARPi, antimetabolites and platinum-based chemotherapy [149]. The FDA has granted fast track designations to prexasertib in platinum-resistant ovarian cancer and endometrial cancer as monotherapy or in combination with low-dose gemcitabine (NCT05548296). As *MYCN* amplification has been shown to increase RS it is considered as a possible additional biomarker for use of CHK1i like prexasertib in neuroblastoma [150]. Prexasertib was tested either as a single agent or in combination with PARPi olaparib in serous carcinoma PDX models and in a panel of ovarian cancer cell lines [141,151,152]. Several phase 1 and 2 clinical trials are ongoing (Table 2). 

SRA 737 (PNT 737, CCT245737) is a novel orally bioavailable selective CHK1i that has shown preclinical activity in *MYC*-amplified models of neuroblastoma [153] and lymphoma [154]. CHK1 inhibition by SRA 737 showed synthetic lethality with loss of B-family DNA polymerase function in lung and colorectal cancer cells [155].

MK-8776 (SCH 900776) a highly selective dual CHK1/2i [156]. This inhibitor was studied as a monotherapy and in combination with gemcitabine in patients with advanced solid tumors in phase 1 of clinical trials [157]. MK-8776 is capable of restoring the sensitivity for chemotherapy drugs in cancer cells that overexpress P-glycoprotein, the ABC transporters which regulate the uptake and efflux of chemotherapeutics [158].

**Table 2:** Current clinical trials of CHK1i.

Compound	Study Phases	Key Indications	References
Prexasertib (LY2606368, ACR-368)	Phase 2 (7 trials)	Different types of tumors, including small cell lung cancer, ovarian cancer, etc.	NCT02735980 (completed) [159] NCT03414047 (completed) NCT02203513 (terminated) NCT02873975 (completed) NCT04095221 NCT04032080 (completed) NCT05548296
	Phase 1 (14 trials)	Different types of solid tumors and leukemias	NCT02778126 (completed) NCT02514603 (completed) NCT03495323 (completed) NCT02860780 (completed) NCT01115790 (completed) NCT03057145 (completed) NCT04095221 NCT02808650 (completed) NCT04023669 NCT05548296 NCT03735446 (terminated) NCT02649764 (completed) NCT02124148 (completed) NCT02555644 (completed)
SRA 737	Phase 1/2 (2 trials)	Advanced solid tumors or non-Hodgkin’s lymphoma	NCT02797964 [159,160] NCT02797977
MK-8776 (SCH 900776)	Phase 2 (1 trial)	Leukemias	NCT00907517 (terminated) [161]
	Phase 1 (2 trials)	Solid tumors, leukemias and lymphomas	NCT00779584 (completed)

The ATRi and especially CHK1i drugs have been extensively developed only in recent years, and available clinical data is relatively limited, compared with approved PARPi. Hematologic and gastrointestinal toxicities remain major hurdles for ATRi and CHKi [162]. More clinical data and basic research would hopefully allow to determine precise markers and indications for these drugs, maximizing the therapeutic window.

### 3.2. PARP Inhibitors

Presently many PARPi such as olaparib, rucaparib, niraparib and talazoparib are approved by the US FDA primarily for *BRCA1/2*-mutated tumors [163,164]. Recent data about clinical trials of these drugs are represented in Table 3. It is pivotal to verify *BRCA1/2* status of patients before therapy due to the role of HR in the potency of PARPi usage. However, even *BRCA1/2*-mutated tumors can manifest resistance to PARPi. There are many ways to perform it such as replicative fork stabilization, HSP90-mediated BRCA1 stabilization and subsequent HR repair [165], or recently researched recruitment of protein complex shieldin: REV7, RINN1, RINN2, and RINN3 (Figure 3). This complex promotes NHEJ-dependent DNA repair by ATM-53BP1-RIF1-REV7 pathway that leads to the development of resistance to PARPi by the HR-independent pathway [166,167].

Some trials indicated that PARPi treatment may be improved by addition of ATRi [168,169]. That being the case, many up-to-date studies suggest that PARPi as single agents or in combination with particular compounds can be sufficiently effective against a wider variety of tumors than was thought before. Numerous clinical trials demonstrate beneficial results of PARPi treatment of pancreatic cancer [170], urothelial carcinoma [171], NCT03397394) and mesenchymal sarcomas [172]. 

Previously, it was demonstrated that PARP inhibition therapy led to an increase of ATR and CHK1 phosphorylation, suggesting that activation of the ATR-CHK1 replication fork protection pathway is one of the main ways to save genome stability in response to PARP shortage. Hence, inhibition of ATR (ceralasertib) or CHK1 (MK-8776) in combination with olaparib led to a considerable synergistic effect that was proved by experiments on HGSOC both in vitro and in vivo [139]. Interestingly, Parmar et al. [141] demonstrated that combination therapy with olaparib and a CHK1i prexasertib was highly effective in models of ovarian and osteosarcoma cancer cells and PDX, resistant to PARPi monotherapy. The mechanism of synergy was associated with *RAD51* depletion and replicative fork destabilization; the best effect was achieved in combination treatment of *RAD51*-mutated cells. Experiments on xenograft models (HGSOC) with *BRCA1* mutations and without them showed that PARPi monotherapy did not cause effect, but models were sensitive to CHK1i prexasertib monotherapy. Combination of PARPi and CHK1i showed increased efficiency, compared with CHK1i monotherapy.

Another growing field for PARPi usage is combination treatment with WEE1 inhibitors such as a novel small molecule adavosertib (AZD1775). It might be connected with the G2/M checkpoint, which is vital for correct DNA repair before mitosis and further cell cycle progress. Inhibition of WEE1 compromises G2 arrest, leading to abnormal exit to mitosis, and accumulation of DNA damage, leading to RS and subsequent cell death. Thus, it was recently demonstrated [173] that WEE1i adavosertib monotherapy of TNBC was effective, inhibiting RAD51-mediated HR DNA repair, and increasing the quantity of γH2AX foci. Experiments on breast cancer cell lines (MDA-MB-231, BT-549) showed synergistic effects with combination of PARPi olaparib and adavosertib. During combinational treatment, the number of DNA-damaged cells was greater than in adavosertib monotherapy; such synergy was caused by HR deficiency that noticeably enhanced PARPi impact. Besides, the same study demonstrated highly efficient synergism of tumor growth inhibition by PARPi and WEE1i combined treatment in human breast cancer xenograft models (MDA-MB-231) without significant toxicity. Similar results were also reported by [174], emphasizing G2-M arrest induced after PARP inhibition by talazoparib. PARP inhibition led to increased expression or phosphorylation of major proteins involved in S and G2 DNA damage checkpoints: Cyclin B1, Rb, WEE1, CDK1, FOXM1, CHK1, CHK2 and ATM. Interestingly, sequential therapy did not reduce the efficiency of PARPi and WEE1i combination for ovarian cancer cells in vitro and in vivo, compared to concurrent inhibition, while reducing toxicity for non-transformed cells.

**Table 3:** Clinical trials of approved PARPi.

Compound	Study Phases	Key Indications	References
Olaparib	Phase 4 (4 trials)	Ovarian cancer (3 trials), prostate cancer (1 trial), metastatic breast cancer (1 trial)	[175,176,177,178]
	Phase 3 (37 trials)	Ovarian cancer (more than 30 trials), breast cancer (13 trials), prostate cancer (4 trials)	
	Phase 2 (more than 200 trials)	Ovarian cancer (more than 30 trials), breast cancer (more than 30 trials), prostate cancer (more than 20 trials), lung cancer (more than 20 trials)	
	Phase 1 (more than 100 trials)	Ovarian cancer (more than 30 trials), breast cancer (more than 20 trials), prostate cancer (12 trials), lung cancer (10 trials)	
Niraparib	Phase 4 (3 trials)	Ovarian cancer (3 trials)	[179,180,181,182]
	Phase 3 (23 trials)	Ovarian cancer (12 trials), fallopian tube cancer (5 trials), prostate cancer (3 trials), breast cancer (2 trials),	
	Phase 2 (more than 100 trials)	Ovarian cancer (more than 30 trials), breast cancer (15 trials), fallopian tube cancer (9 trials)	
	Phase 1 (62 trials)	Ovarian cancer (19 trials), breast cancer (13 trials), prostate cancer (7 trials)	
Rucaparib	Phase 3 (8 trials)	Ovarian cancer (4 trials), fallopian tube cancer (4 trials), prostate cancer 2 trials)	[183,184,185,186]
	Phase 2 (40 trials)	Ovarian cancer (8 trials), prostate cancer (7 trials), breast cancer (4 trials),	
	Phase 1 (24 trials)	Ovarian cancer (7 trials), breast cancer (4 trials), prostate cancer (4 trials)	
Talazoparib	Phase 3 (5 trials)	Ovarian cancer (2 trials), breast cancer (1 trial), prostate cancer (1 trial)	[187,188,189,190]
	Phase 2 (64 trials)	Breast cancer (17 trials), prostate cancer (8 trials), ovarian cancer (4 trials)	
	Phase 1 (51 trials)	Breast cancer (14 trials), prostate cancer (5 trials), ovarian cancer (4 trials)	

### 3.3. WEE1 and PKMYT1 Inhibitors

WEE1 and PKMYT1 (MYT1) are two protein kinases that regulate activity of CDK complexes through inhibitory phosphorylations. WEE1 inhibits activity of CDK2 at the both G1/S and G2/M transitions, while PKMYT1 is active only in the G2/M checkpoint. Both are rarely mutated in cancers and in tumors with high levels of RS they act as oncogenes, protecting cells from excessive DNA damage. Both are overexpressed in many hematological and solid tumors [191]. 

Currently, the most commonly investigated WEE1i is a small molecule AZD1775 (adavosertib, Table 4). Presently it is in trials as an anticancer drug for different types of solid tumors even in pediatric patients [192]. The most recent studies involved in phase I or phase 2 of clinical trials are examining WEE1i for the treatment of pancreatic, gastric, head and neck, breast, ovarian, and other tumors [193]. Many therapies with WEE1i frequently include combinations with other drugs that induce RS, such as carboplatin [194], gemcitabine [195], and PARPi like olaparib [196].

WEE1 inhibition by adavosertib in tumor cells causes acceleration of cell cycle promotion by activation of CDK2 that, in turn, leads to RS, DNA aberrations and further cell death. Other research by Lindemann and colleagues [197] suggested that DNA aberrations and RS, caused by WEE1 inhibition, could be useful for cancer treatment under conditions of DNA repair disorder. Combined incubation with Rad51 inhibitor B02 and adavosertib manifested notable synergism, with increased markers of DNA damage (γH2AX) and RS (pRPA32), and levels of cell death, than in monotherapy. As was demonstrated in previous works [198], CHK1 phosphorylation level was decreasing, while CDK1 activity was rising that, as a result, caused accumulation of RS, indicating DSB repair shortage. Interestingly, in HPV-positive lines, an increase of p53 level was observed that can be connected with activity of E6 or E7 oncogenes [199], although p21 level was increased irrespective of HPV status in response to combination of B02 and adavosertib. In vivo experiments in mouse models of oral tongue cancer have shown that HPV-negative tumors were not sensitive to B02 and adavosertib mono- or combined therapy. In HPV-positive mice, drugs’ combination significantly inhibited growth of the tumor and substantially increased animal survival rate [197]. These findings are very important for the therapeutic aims due to the role of Rad51 in patients’ survival rate [200].

P53 status is found to play a significant role in WEE1i performance. Thus, *TP53*-depleted HNSCC cells demonstrate remarkable accumulation of SSB and DSB DNA damage markers and PARP1 cleavage in response to adavosertib, compared with *TP53* WT (wild type) cells. Interestingly, the number of 53BP1 foci, which is an important marker of DNA damage, was lower in *TP53* knockdown cells, however most of the 53BP1-positive cells did not express γH2AX. It can be considered as a mismatch between 53BP1 foci localization and DSBs [201]. Furthermore, previous study investigated that WEE1i sensitized *TP53*-mutated mouse xenografts to cisplatin exposure, indicating potency of adavosertib as an effective supplemental drug for *TP53*-mutated tumors therapy [202]. Data from the previously mentioned article [138] revealed that adavosertib and ATRi ceralasertib treatments slowed progression of the replication fork and increased origin firing. WEE1i led to activation of the ATR–CHK1 pathway and decrease of CHK1 and ATM protein expression after 24 h. However, the combination of adavosertib and ceralasertib was not effective in vivo, resulting in tumor regressions comparable to the adavosertib single-agent.

*PKMYT1* is overexpressed in a number of tumors with markers of RS and PKMYT1 inhibitors (PKMYT1i) are effective in preclinical in vitro and in vivo models. PKMYT1 inhibition was effective in *MYCN*-amplified neuroblastomas, but not neuroblastomas without amplification [203]. Blocking PKMYT1 activity was effective in eradication of *CCNE1*-amplified ovarian cancer cells, but not cell lines without amplification through preventing completion of DNA synthesis and increasing the rates of premature mitotic entry [44]. Notably *PKMYT1* is overexpressed in *CCNE*-amplified ovarian carcinoma [204], and currently PKMYT1i RP-6306 is in clinical trials in this setting (Table 4). 

Additionally, PKMYT1 and WEE1 inhibition synthetically eradicates cancers with high levels of RS such as glioma [205] and HGSOC, relatively sparing normal tissues or cancers with lower levels of RS [206]. As WEE1i are hindered by toxicity, the authors consider such combinations more selective for therapy. As PKMYT1, but not WEE1, is more important for G2/M transition during checkpoint recovery [207] its inhibitors could be important for a number of combinational therapies aimed at RS.

**Table 4:** Clinical trials of WEE1 and PKMYT1 inhibitors.

Compound	Study Phases	Key Indications	References
Adavosertib (AZD1775)	Phase 2 (32 trials, including 7 terminated and 1 withdrawn)	Solid tumors, harboring *CCNE1* amplification, ovarian (2 studies), neuroblastoma, medulloblastoma, and rhabdomyosarcoma	[194,195,208,209,210,211,212,213,214,215]
	Phase 1 (34 trials, including 6 terminated and 1 withdrawn)	HNSCC, uterine cancers, TNBC, pancreatic cancer, acute myeloid leukemia, glioblastoma	[192,193,216,217,218,219,220,221,222,223,224,225,226,227,228]

### 3.4. CDK Inhibitors and RS

Cyclin-dependent kinases in complexes with their Cyclins regulate several critical processes in the cell. The better-known group, consisting of CDK1-6, primarily controls the transition through stages of the cell cycle, while other CDKs, such as CDK7, CDK8/19, CDK9 and CDK12/13 are mainly involved in transcription [229]. Interestingly both groups of CDKs are implicated in RS and DDR, as proper alignment of transcription and cell cycle transition is critically important to the proper replication process (Figure 3). As inhibitors of CDKs are approved by the regulator or are investigated in clinical trials, more studies focus on their impact on tumors with increased RS (Table 5).

**Table 5:** Clinical trials of CDK2 inhibitors in tumors with RS markers.

Compound	Study Phases	Key Indications	References
BLU-222	Phase 1/2 (1 trial)	Solid tumors, including *CCNE1*-amplified, ovarian carcinoma, breast cancer, endometrial and gastric cancer	NCT05252416
INX-315	Phase 1/2 (1 trial)	Solid tumors, including breast cancer who progressed on a prior CDK4/6i regimen, and *CCNE1*-amplified solid tumors	NCT05735080
PF-07104091	Phase 1/2 (2 trials)	Small cell lung cancer, ovarian cancer, breast cancer	NCT04553133NCT05262400
ARTS-021	Phase 1/2 (1 trial)	*CCNE1*-amplified solid tumors	NCT05867251
INCB123667	Phase 1 (1 trial)	Solid tumors	NCT05238922

CDK1 is the Cyclin-dependent kinase essential for cell cycle progression in S, G2 and M phases of the cell cycle and can also replace other Cyclin-dependent kinases in many models [19,230]. CDK1 inhibition compromises the BRCA1-dependent ATR and ATM DDR in the S phase, increasing sensitivity to DNA damaging agents [231]. Compromised CDK1 activity also leads to increased sensitivity to PARPi [232]. On the other hand, CDK1 inhibition is an important mechanism, limiting transcription-replication conflicts [17].

As discussed above, CDK2/Cyclin E is responsible for G1/S entry by phosphorylating Rb and components of the replication machinery such as CDC6, treslin and RECQL4, as well as CDT1 and MCM complex components [35]. Amplification of the *CCNE1* is common in many cancers, especially in breast and ovarian carcinomas. Increased activity of CDK2/Cyclin E in these tumors is known to increase RS and genetic instability via several mechanisms such as shortening of the G1 phase and aberrant origin licensing [35]. Although *CCNE*-amplified tumors are more prone to RS they remain hard to treat using standard DNA-damaging chemotherapy regimens, especially in ovarian cancer. Several studies revealed, counterintuitively, that CDK2 participates in DNA repair [7,233], and *CCNE*-amplified ovarian carcinomas rely on CDK2 for DNA repair through homologous recombination, its inhibition compromises replication fork repair [7]. The same study also demonstrated that Cyclin E1 is present at the stalled forks, probably participating in repair.

CDK4/6 inhibitors (palbociclib, ribociclib, abemaciclib) are known as effective drugs for breast cancer treatment irrespective of the p53 status [234,235]. Long-term G1 arrest induced by palbociclib caused RS that was a reason for significant cell proliferation decrease due to transition to senescent state of *TP53* WT cells or, in the absence of p53, by causing cells to undergo mitotic catastrophe, resulting in DNA damage [15]. This difference in cells’ fate depended on the level of p21— one of the main proteins involved in transition to a senescence condition. Thus, in WT cells, p53-induced p21 level rise, while in *TP53* KO (knockout) cells induction was absent. Effect of palbociclib for *TP53* WT and especially *TP53* KO was ATR-dependent: ATR inhibition after CDK4/6i 7 days treatment demonstrated an increase in the number of fragmented nuclei that is a consequence of chromosome segregation errors. These data suggested that prolonged CDK4/6 inhibition led to a shortened period of replication and increased the number of cells, which enter mitosis prematurely.

Transcriptional CDKs are also implied in RS regulation. CDK8 and CDK19 are two homologous kinases which regulate initiation of transcription as part of the Mediator complex. They are required for transcription activated by a number of transcription factors, such as STATs, SMADs, beta-catenin, p53 and others [236]. Similarly to CDK2, CDK8/19 have a dual role in response to RS. One article demonstrates that CDK8/19 deletion decreases RS due to replication-transcription conflicts [237], and CDK8/19 activity is required for sensitivity to ATRi and CHK1i. On the other hand, in uterine fibroids inhibition of CDK8/19 increased the number of stalled replication forks and markers of RS, including ATR phosphorylation. This phenotype was also dependent on R-loop formation [238]. Additionally, at least, in certain cancers such as prostate carcinoma inhibition of CDK8/19 led to increased ATR-dependent RS and DNA damage by inducing aberrant G1/S transition [14]. Another recent study has demonstrated a similar increased G1/S transition in chronic myelogenous leukemia [239]. CDK8/19 is also required for normal origin firing during replication by interacting with the MTBP (Mdm2 p53 binding protein) complex with Treslin, and its inhibition leads to an increase in the number of fragile metaphase chromosome sites [240]. It is possible that similarly to CDK2, CDK8/19 participates in ATR-alternative repair pathways, during RS, as DNA-repair proteins such as BRCA2 and MDC1 were identified as CDK8/19 substrates [241], and inhibition of CDK8/19 increased activity of DNA-damaging agents [242].

While CDK8/19 are involved in initiation of transcription, CDK12 and CDK13 regulate transcription termination and splicing. CDK12/13 are involved in transcriptional regulation of a number of DDR genes such as *BRCA1*, *ATR* and *FANC1* [243]. CDK12 expression is required for expression of core replication genes, and its inhibition delays G1/S transition and increases in the number of chromosomal aberrations. This replication-dependent DNA damage is caused by reduced processivity of RNA polymerase II in long poly-(A)-signal-rich genes [244]. CDK12 was implicated in sensitivity to PARPi [13] as well as survival of ovarian cancer cell lines irrespective of synergy with PARPi [245].

### 3.5. Biomarkers of Replication Stress and Response to RS-Inducing Drugs

Selection of patients who would benefit from a particular targeted therapy is one of the most critical aspects of modern cancer treatments. This is especially crucial for drugs that induce RS or target components of RS-response as they are highly active and toxic, as well as maximizing the therapeutic window remains a particular challenge. As such selection of patients with *BRCA1/2* mutations was crucial for the success of PARPi and this biomarker has remained the main guiding principle for this therapy [246]. Nevertheless, adoption of such single markers for other RS-related treatments proved to be a difficult task, due to the complexity of the RS-phenotype and many of the players involved. Currently, two types of biomarkers are used to predict the response to RS-targeted drugs—one is mutations and overexpression of oncogenes and the other are alterations in DDR pathways and high expression of DDR proteins. Amplification, point mutations and high levels of *CCNE1*, *MYC* and *KRAS* fall in the first category. Amplification of *CCNE1* is used as an eligibility marker in trials for CDK2 inhibitors (NCT05238922, [247]), WEE1i [215], and PKMYT1i [248]. The second category uses staining for phospho-RPA32, RAD51, γH2AX or TP53BP foci, as well as alterations of ATM and RAD51C pathway (such as *TP53* mutations, low levels of RAD51, etc.) [249]. *ATM* defects are frequently used for selection of patients for ATRi therapy, due to ATM being not only a biomarker for RS-high tumors, but as a synthetically lethal pair for ATR [250]. Mutations of *TP53* and high level of CHK1 are used for selection of patients for CHK1 trials [251]. Often scores involving several validated markers are used. For example, a score defined by any changes “*CCNE1* amplification, *RB1* two-copy loss, *CDKN2A* two-copy loss, *KRAS* amplification, *NF1* (gene of transcription factor Neurofibromin 1) mutations, *ERBB2* (gene of HER2) amplification, *MYC* amplification, and *MYCL1* (gene of transcription factor L-Myc) amplification” was used as predictor for sensitivity of HGSOC ovarian cancer to gemcitabine and ATRi [45]. In RS-high cancers alterations in oncogenes with the DDR response are often correlated, validating a link between them—such as expression of *CCNE* and high levels pRP plus γH2AX staining [252]. 

A more complex approach relies on using complex scoring algorithms and/or expression signatures to classify tumors as RS-high and RS-low. One such score (repstress), using a transcriptional profile, was developed based on data from cell lines and then validated in clinical samples. This score correlated with γH2AX staining and expression of RS-related genes such as *TIMELESS*, *CLSPN*, *TOP2A*, *FANCD2* and others, and high levels of oncogenes—*CDC25A*, *CCNA2* and others. Additionally, the repstress score predicted response to RS-therapies, including ATRi [253]. Similar complex algorithm using multi-omics data was developed for prostate cancer [254]. While potentially much more precise for patient selection and predicting response to RS-targeting drugs such methods are more expensive and could be challenging in routine clinical practice.

## 4. Conclusions and Future Directions

Tumors with increased RS are among the hardest to treat. Basal type breast cancer and platinum-resistant ovarian carcinomas have dismal prognosis, compared to same localizations, which don’t have elevated RS [45,253]. In recent years drugs that target cancers with increased RS and inhibited DNA damage response started to enter clinical trials and even progressed to the clinic (PARPi for *BRCA1/2*-mutated cancers). Targeted inhibitors were developed against almost every link of DDR to RS, and key proteins which safeguard cancers against replication catastrophe. These approaches have yielded a hope that survival in some of the most aggressive cancers can be radically improved. Nevertheless, the road to introduction of these medications into the clinic remains difficult, with high toxicity and small therapeutic windows leading to termination of a number of programs, and even putting some RS targets into doubt [162,255]. Basic research has demonstrated that sensitivity of cancers to inducers of RS is highly dependent on markers, with most clinical development programs recently focusing on a narrower group of populations with such markers as *CCNE* and *MYC* amplifications, *RB1* and *TP53* mutations and others. Two future directions may improve the efficiency of targeting high RS cells. First, a number of inhibitors targeting more specific targets, such as Rad51 [256], can limit toxicities, seen for more broad inhibitors, such as ATRi. Second, as more clinical data becomes available, improved markers and even gene signatures [253,257] can allow precise targeting of cancers most susceptible to particular inhibitors [258] and predicting responses [254]. More sophisticated dosing schedules, with sequential treatment with DNA damaging agents and RS inducers are also a promising development [259].

## Data Availability

No new data were created.

## Abbreviations

Anti-PD-L1	anti-programmed death ligand 1
ATM	ataxia telangiectasia mutated
ATMi	ATM inhibitor(s)
ATR	ataxia telangiectasia and Rad3 related protein
ATRi	ATR inhibitor(s)
BER	base excision repair
CDK(s)	Cyclin dependent kinase(s)
CHK	checkpoint kinase
CHK1i	CHK1 inhibitor(s)
DLBCL	diffuse large B-cell lymphoma
DDR	DNA damage response
DSB(s)	double-strand DNA break(s)
HGSOC	high-grade serous ovarian cancer
HNSCC	head and neck squamous cell carcinoma
HPV	human papilloma virus
HR	homologous recombination
KO	knockout
LMW-E	low-molecular-weight Cyclin E
NHEJ	non-homologous end-joining repair
NSCLC	non-small cell lung cancer
PARP	poly (ADP-ribose) polymerase
PARPi	PARP inhibitor(s)
PDAC	pancreatic ductal adenocarcinoma
PDX	patient-derived xenograft model(s)
PKMYT1i	PKMYT1 inhibitor(s)
RPA	replication protein A
RS	replication stress
SSB	single-strand DNA breaks
TNBC	triple-negative breast cancer
WEE1i	WEE1 inhibitor(s)
WT	wild type
